# MTP18 is a Novel Regulator of Mitochondrial Fission in CNS Neuron Development, Axonal Growth, and Injury Responses

**DOI:** 10.1038/s41598-019-46956-5

**Published:** 2019-07-23

**Authors:** Alexander Kreymerman, David N. Buickians, Michael M. Nahmou, Tammy Tran, Joana Galvao, Yan Wang, Nicholas Sun, Leah Bazik, Star K. Huynh, In-Jae Cho, Tomasz Boczek, Kun-Che Chang, Noelia J. Kunzevitzky, Jeffrey L. Goldberg

**Affiliations:** 1 Byers Eye Institute and Spencer Center for Vision Research, Stanford University, Palo Alto, CA 94303 USA; 2 University of Miami Miller School of Medicine, Miami, FL 33136 USA; 3 University of California, San Diego, CA 92093 USA

**Keywords:** Axon and dendritic guidance, Regeneration and repair in the nervous system, Molecular neuroscience, Cell death in the nervous system

## Abstract

The process of mitochondrial fission-fusion has been implicated in diverse neuronal roles including neuronal survival, axon degeneration, and axon regeneration. However, whether increased fission or fusion is beneficial for neuronal health and/or axonal growth is not entirely clear, and is likely situational and cell type-dependent. In searching for mitochondrial fission-fusion regulating proteins for improving axonal growth within the visual system, we uncover that mitochondrial fission process 1,18 kDa (MTP18/MTFP1), a pro-fission protein within the CNS, is critical to maintaining mitochondrial size and volume under normal and injury conditions, in retinal ganglion cells (RGCs). We demonstrate that MTP18’s expression is regulated by transcription factors involved in axonal growth, Kruppel-like factor (KLF) transcription factors-7 and -9, and that knockdown of MTP18 promotes axon growth. This investigation exposes MTP18’s previously unexplored role in regulating mitochondrial fission, implicates MTP18 as a downstream component of axon regenerative signaling, and ultimately lays the groundwork for investigations on the therapeutic efficacy of MTP18 expression suppression during CNS axon degenerative events.

## Introduction

Mitochondrial fission-fusion and the associated changes in mitochondrial size have been shown to play important and diverse roles in neurobiology, such as the maintenance of axonal homeostasis or axon degeneration in development, aging, and disease^1–3^. The influence of mitochondrial fission-fusion on ATP production, redox state, and calcium homeostasis have been heavily investigated in axonal health and dysfunction, but to what degree does the fission or fusion state of mitochondria influence axonal growth? With only a few fission-fusion regulators currently identified and over a 1,000 proteins found associated with or in neuronal mitochondria^4–6^, it is possible that additional fission-fusion proteins exist with roles in axonal growth or regeneration.

Studies have implicated mitochondria as integral components in axon regeneration^7–9^, with some evidence suggesting a role of mitochondrial fission-fusion in orchestrating these events. Adding support to a fission-fusion role in axonal growth, we have previously shown that suppressing fission or increasing mitochondrial fusion in RGCs, visual system neurons subject to permanent axon loss after injury, improved axonal growth despite the presence of growth suppressive molecules, such as chondroitin sulfate proteoglycans (CSPGs)^10^. In addition, KLF4 and -6, which differentially regulate CNS neurons’ intrinsic axon regenerative capacity^11,12^, have been shown to differentially regulate mitochondrial size^10^. Other prominent axonal growth regulators such as BDNF^13^, NGF^14,15^, BCL-2^16,17^, PTEN^18–20^, and Nogo^21,22^, have also been shown to directly or indirectly influence mitochondrial size and/or associated mitochondrial activities. Thus, data suggest that mitochondrial fission-fusion might be a key downstream mediator of axonal growth signaling factors and regenerative responses.

Here we expand on these observations by presenting evidence for a mechanism in which axonal growth promoting and suppressing factors, KLF7 and -9, respectively, regulate mitochondrial fission and fusion via the expression of a nuclear-encoded mitochondrial gene, MTP18. MTP18 has not been well-studied in neurobiology, but it is among a set of new genes shown to regulate fission^23,24^. First identified and investigated within the cancer field using HeLa and COS-7 cell lines, MTP18 was demonstrated as a necessary component for promoting mitochondrial fission together with dynamin related protein-1 (Drp-1) and Mitochondrial fission 1 protein (Fis1)^23,24^. However, unlike Fis1 and Drp-1 which participate in canonical GTP-dependent outer mitochondrial membrane (OMM) fission, MTP18 localizes within the mitochondria and is thought to coordinate a novel process of inner mitochondrial membrane (IMM) fission with OMM fission^23^. MTP18 was also initially shown to be expressed at undetectable/low level in the CNS, in spinal cord and brain extracts^24^. However, we find that MTP18 is expressed within the CNS and is detectable at both the RNA and protein level within retinas and RGCs. As a result, here we aim to identify and possibly expand MTP18’s pro-fission role within CNS neurons, and present data implicating this novel protein as a prominent regulator of both mitochondrial size and axonal growth during CNS development and injury conditions.

## Results

### KLFs Promote the Expression of Mitochondrial Genes

**Figure 1:**
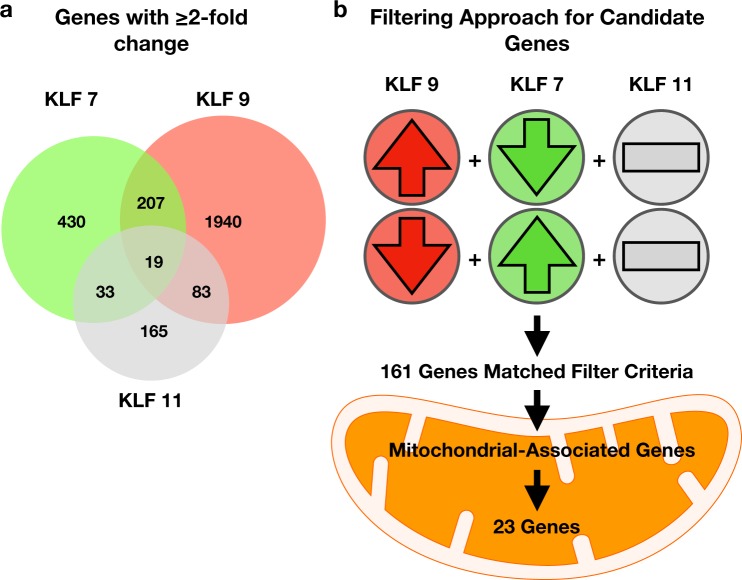
Microarray data collected from KLF expression experiments in RGCs. (**a**) KLF regulated genes expressed greater than or equal to 2–fold in a positive or negative direction. Data collected from lentivirus mediated KLF7, -9 or -11 expression in cultured RGCs. (**b**) Filtering scheme to identify axon growth regulating genes from a list of 2 fold changing genes. Genes were grouped based on opposing outcomes of KLF expression on RGC axon growth enhancement/suppression. Arrows represent a directions fold change and bars represent no fold change. Up/down regulations were considered when changes were >2 fold with both KLFs, or slightly relaxed criteria of 2 fold change with one KLF and >1.5 with the other KLF. In all scenarios the KLF11 regulation of a particular gene had to be <1.5. A total of 163 genes met these criteria, of which 23 were considered mitochondrial associated based on gene ontology analysis. All fold changes were identified relative to lentivirus mCherry controls, and data were generated from N = 5 or more repeat RGC isolation, virus treatments, and microarray chips. Data analyzed with GenSpring 12.5–GX software, significantly expressed genes identified by a Oneway ANOVA and Welch test, p (Corr) cut-off of 0.05. See Supplementary Dataset 1, for raw, filtered, and candidate gene lists.

To investigate whether mitochondrial dynamics are involved in CNS axonal growth, we ask whether mitochondrial fission-fusion genes are being regulated by a set of growth suppressive or regenerative transcription factors, the KLFs. KLFs consist of a family of 17 factors, many of which have suppressive and axonal growth promoting potential *in vitro* and *in vivo*^11^. In this study, we focused on mitochondrial gene regulation by the two most promising KLFs, KLF7 and -9, which have been shown to promote axon regeneration both in the corticospinal tract and optic nerve^12,25^. To gather information on the underlying genetic regulations responsible for KLF-mediated axon regeneration, we analyzed gene expression microarray data from lentivirus mediated expression of KLF7 and -9 and in postnatal RGCs. We found that together, KLF7 and -9 regulate the mRNA expression of more than 2000 different genes by greater than 2 fold (Fig. 1a).

Given the large amount of gene expression changes stimulated by KLF expression, we filtered KLF-regulated genes for changers that might be relevant to axon growth and subsequently genes associated with mitochondria. To accomplish this, we used our previously validated knowledge of the phenotypic effects of KLFs on axon growth both *in vitro* and *in vivo*. More specifically, genes were considered as axon growth regulating candidates when expressed in opposing directions by KLF9, an axon growth suppressing factor, and KLF7, an axon growth enhancer, but were not significantly up/down regulated by KLF11, shown not to regulate axon growth (Fig. 1b). Of note, although this filtering method does not capture targets of individual KLFs and thus may miss potentially important target mitochondrial gene candidates, it narrows the candidate list on the premise that the coordinated regulation of axon growth by many KLFs may be regulated by common target genes. In addition, this filtering method has been proven to generate other gene candidates involved in KLF signaling and axon growth^26^. Using this method, we identified a list of 161 axon growth regulating candidate genes of which approximately 15% (23 genes) were mitochondrial associated candidates, and which contained a greater than 2 fold enrichment for previously validated axonal/neuronal projection regulated genes, based on GO enrichment terms (GO Ontology Consortium^27,28^). Overall, our filtering method suggests that the 23 mitochondrial candidate genes (Table 1) might be downstream gene expression targets for KLFs, directly involved in axon growth or part of a network of genes involved in axon growth.

**Table 1:** Mitochondrial-associated gene candidates that are also associated with axon growth regulation.

Gene Symbol	KLF7	KLF9	KLF11
Abcd2	−1.59 (±0.17)	9.15 (±0.11)	1.13 (±0.15)
Adck3	−1.53 (±0.08)	13.57 (±0.05)	−1.37 (±0.09)
Akr7a2	−1.53 (±0.08)	2.64 (±0.05)	1.12 (±0.04)
Aldh5a1	−2.91 (±0.21)	2.19 (±0.06)	1.07 (±0.15)
Apex1	−1.82 (±0.13)	2.76 (±0.06)	1.37 (±0.067)
C19orf12	−2.65 (±0.18)	2.84 (±0.04)	1.37 (±0.05)
Dimt1	−1.53 (±0.13)	2.29 (±0.03)	1.21 (±0.07)
Epha4	−2.39 (±0.16)	2.55 (±0.07)	−1.35 (±0.17)
Gad1	−1.87 (±0.18)	5.25 (±0.2)	−1.12 (±0.22)
Hrsp12	−1.52 (±0.09)	2.41 (±0.05)	1.23 (±0.03)
Itgax	−2.46 (±0.14)	3.53 (±0.3)	1.02 (±0.27)
Lpin1	−1.69 (±0.09)	2.10 (±0.04)	1.40 (±0.03)
Macrod1	−1.68 (±0.05)	2.51 (±0.13)	1.20 (±0.10)
MTP18	−1.75 (±0.19)	3.40 (±0.04)	1.18 (±0.036)
Nceh1	−2.04 (±0.12)	2.49 (±0.04)	1.07 (±0.03)
Pdk2	−1.87 (±0.12)	17.89 (±0.14)	1.11 (±0.07)
Pecr	−1.67 (±0.34)	8.50 (±0.11)	1.40 (±0.15)
Phyhip	−1.67 (±0.39)	3.57 (±0.19)	1.10 (±0.09)
Pnpo	−1.51 (±0.1)	3.34 (±0.04)	−1.04 (±0.06)
Ppargc1a	−3.72 (±0.39)	3.77 (±0.21)	1.27 (±0.53)
Tmtc1	−2.22 (±0.14)	1.56 (±0.07)	1.10 (±0.13)
Vamp1	−1.53 (±0.05)	2.80 (±0.05)	−1.01 (±0.03)
Vars2	−1.51 (±0.3)	2.55 (±0.03)	1.05 (±0.19)

Fold change presented relative to mCherry controls. Data represented as mean expression ± SEM values (N = 5 or more independently repeated experiments).

### Novel fission gene MTP18 is regulated by KLF expression

**Figure 2:**
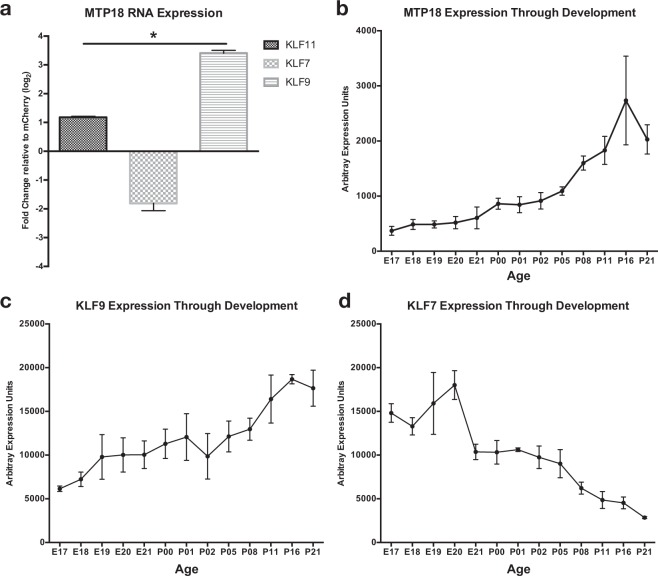
MTP18 expression is regulated in RGCs by KLFs and through development. (**a**) MTP18 mRNA expression after lentivirus mediated KLF 11, -7 and -9 expression, in P4 isolated RGCs, relative to mCherry expressing controls (N = 5 or more independently repeated experiments). Data presented as mean expression, + SEM, identified using multiple microarray chips (significance ascribed by one-way ANOVA and Tukey multiple comparison, *p ≤ 0.05). Relative change in mRNA expression of (**b**) MTP18, (**c**) KLF9, and (**d**) KLF7, in isolated RGCs from E17-P12 by microarray. All groups are N = 3 or more independently repeated experiments. All Error bars represented as ± SEM.

We then focused on candidate mitochondrial genes which might explain our previous finding that suppressing fission (or increasing fusion) leads to improved axon growth^10^. Using these criteria, we asked whether mitochondrial fission-fusion mechanisms also underlie the axon suppressing/enhancing activity of KLF7 and -9. Pertinent to our previous findings, we found that axon growth-promoting KLF7 decreased, and growth-suppressing KLF9 significantly increased, the genetic expression of mitochondrial fission process 1,18 kDa (MTP18), a positive regulator of mitochondrial fission and the only direct regulator of mitochondrial size in our candidate list (Table 1, graphed in Fig. 2a). In addition, we find that MTP18 mRNA increases during developmental time points in which axon growth potential is lost^29^ and KLF7 expression decreases while KLF9 increases (Fig. 2b–d), adding additional support for KLF regulation of MTP18 and a potential axon growth suppressive role for MTP18.

**Figure 3:**
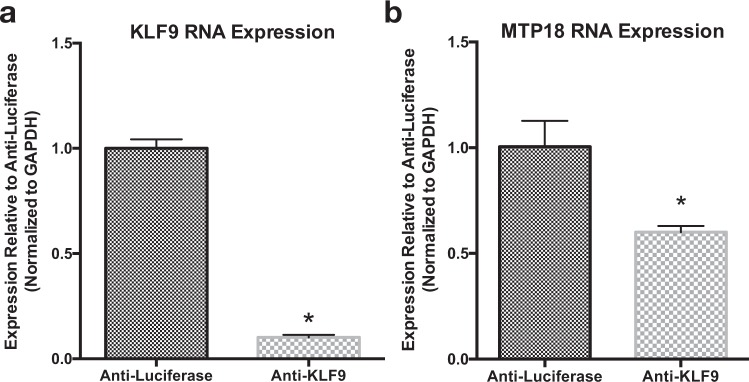
MTP18 expression is regulated downstream of KLF9 expression. (**a**) KLF9 mRNA expression after AAV2 mediated expression of shRNAs targeting KLF9 (Anti-KLF9) and luciferase (Anti-luciferase) mRNA in cultured P4 RGCs. (**b**) MTP18 mRNA expression in P4 RGCs after AAV2 mediated Anti-KLF9 or Ant-luciferase vector expression. All data captured by QRT-PCR, normalized to GAPDH and expressed as mean expression relative to Anti-luciferase (N = 3 replicate treatments, significance ascribed by Student’s t-test,*p ≤ 0.05). All error bars represent + SEM.

However, since KLF9 and MTP18 levels both naturally rise at developmental time points in which KLF9 overexpression data also show an increase in MTP18 expression, it’s possible that our data present a developmentally correlated rather than KLF mediated MTP18 expression change. Of note, this is not likely in RGCs treated with KLF7 expression virus, since MTP18 expression is suppressed despite the presence of a natural increase in levels of MTP18 in untreated cells. As a result, we tested the effect of decreased KLF9 expression on MTP18 mRNA levels. Indeed, our data show that knocking down of KLF9 (Fig. 3a) leads to decreased MTP18 expression in cultured P4 RGCs (Fig. 3b), providing correlative evidence of a mechanism in which KLFs regulate downstream MTP18 expression. Furthermore, since our data suggests that KLF9 is a more potent at regulating RGC axonal growth than KLF7, we focused the rest of our study on the effects of KLF9 signaling onto MTP18 in axonal growth.

### MTP18 and KLF9 regulate mitochondrial size in RGCs

**Figure 4:**
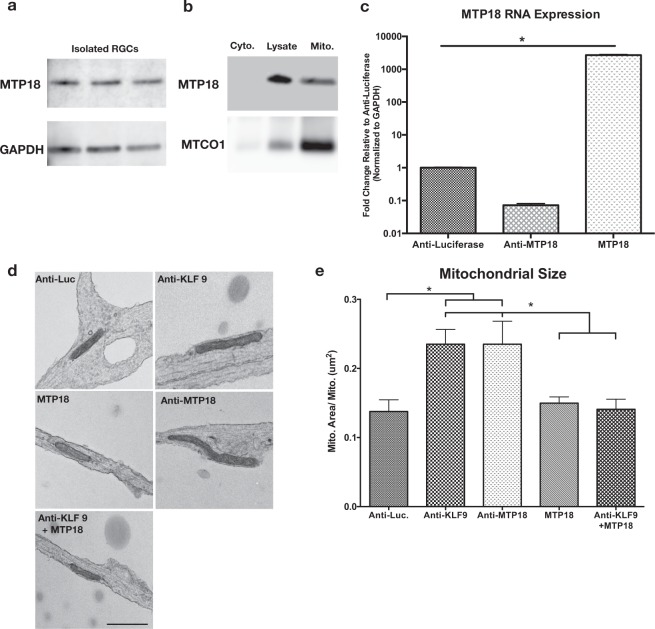
MTP18 is a mitochondria associated protein that regulates mitochondrial size in RGCs. (**a**) Western blot of RGC lysates from 3 replicate RGC purifications probed for MTP18 and GAPDH as a loading control. RGCs were purified from P4 rat retinas. Blots were cut, individually probed with antibodies and then imaged. (**b**) Western blot of a mitochondrial enriched fraction (mito.) cytosolic fraction (cytosolic), and crude total cell lysate fraction (lysate) from adult rat retinas. Samples probed using an MTP18 antibody and Cytochrome c oxidase subunit 1 antibody (MTCO1), to validate mitochondrial enrichment. Images were captured from the same blots, sequentially probed with antibodies. (**c**) MTP18 mRNA expression after AAV2 mediated expression of Anti-luciferase (Anti-Luc), Anti-MTP18, and MTP18 expression vectors in P4 RGCs. Data captured by QRT-PCR, normalized to GAPDH and expressed as mean expression relative to Anti-luciferase (N = 3 replicate treatments, significance ascribed by one-way ANOVA and Tukey multiple comparison, *p ≤ 0.05). (**d**) TEM images of mitochondria in P4 RGCs neurites treated with indicated AAV2 viruses or combinations of virus (scale bar 1 µm). (**e**) Mean area of mitochondria identified in P4 RGC neuritis are graphed for each representative AAV2 treatment group, N ≥ 15 de-identified neurite images selected from 3 replicate viral treatments per group (significance determined by one-way ANOVA and Tukey multiple comparison, *p ≤ 0.05). All error bars represent + SEM. Full length blots are available in Supplemental Fig. 3.

MTP18 is a recently discovered IMM-localized mitochondrial protein shown to promote fission and possibly to regulate cristae organization^23,24^. However, there is no data on whether MTP18 regulates mitochondrial networks within neurons, as investigations on MTP18 have been limited to cancer cell lines with data suggesting that MTP18 mRNA expression is low/non-detectable within the brain or spinal cord^24^. Yet, we find that MTP18 expression is detectable within the visual branch of the CNS, and specifically show MTP18 mRNA expression in retinal ganglion cells along with a confirmation at the protein level and mitochondrial association within retinas (Fig. 4a,b).

To see if MTP18 was also capable of directly regulating the size of mitochondria in CNS neurons, we created vectors and packaged them in Adeno Associated Virus-Serotype 2 (AAV2) viruses to express or knock down MTP18 in isolated and cultured postnatal day 4 (P4) RGCs, and confirmed expression/knockdown by these viruses using qPCR. The knockdown viral constructs were designed to achieve efficient knockdown via the expression of 4 different concatenated shRNAs targeting MTP18 or Luciferase, constructed as previously described^25,30^. Viral efficiency is evident with the reduction of MTP18 mRNA levels by approximately 90% in anti-MTP18 treated RGCs or the increase in MTP18 levels by over 2000% with MTP18 expression-treated RGCs, as compared to anti-luciferase virus controls (Fig. 4c). AAV2 transduced RGCs were then processed for TEM and subsequently characterized for mitochondria size, as a function of individual mitochondrial area (Fig. 4d). Analysis of mitochondria within axons of cultured RGCs revealed that mitochondrial size increased with MTP18 knockdown, compared to luciferase knockdown control plasmids (Fig. 4d,e). Furthermore, reduced KLF9 expression, which we show downregulates the expression of MTP18, was found to also increase mitochondrial size to the same levels as those identified in MTP18 knockdown treatments (Fig. 4d,e). MTP18 expression alone did not decrease mitochondrial size, as compared to luciferase controls. However, in the presence of MTP18 expression, KLF9 knockdown was not able to augment mitochondrial size beyond controls or MTP18 expression treated RGCs. Thus, our data show that while both KLF9 and MTP18 are capable of regulating mitochondrial morphology in CNS neurons, with knockdown of either MTP18 or KLF9 lending to similar mitochondrial changes, MTP18’s influence over mitochondrial activity is dominant over KLF9 and taken together with expression data is likely downstream of KLF9 signaling.

### MTP18 expression increases after optic nerve crush and suppresses mitochondrial size

**Figure 5:**
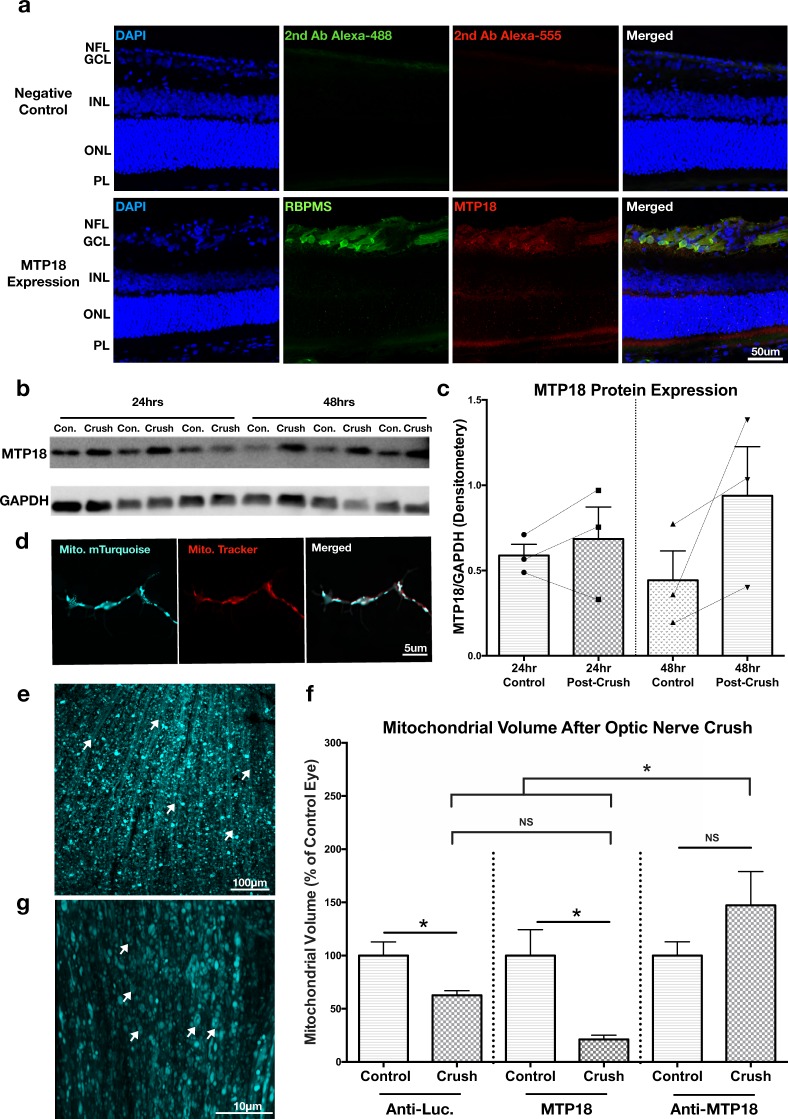
MTP18 expression increases after optic nerve crush injury and is an important component of the acute fission response after injury. (**a**) Immunofluorescence detection of MTP18 in sectioned adult retinas (P40). Top panels are negative controls, stained with secondary antibodies only. Bottom panels were stained with MTP18 and RBPMS, to identify RGCs. DAPI was used to locate the nerve fiber (NFL), ganglion cell (GCL), inner nuclear (INL), outer nuclear (ONL), and photoreceptor layer (PL). Strong MTP18 immunoreactivity was present within the NFL, GCL, and PL. (**b**) Western blot of P40 retina lysates, collected 24 and 48 hrs after optic nerve crush, contralateral-uncrushed eyes served as control samples for each replicate (N = 3 per time point). Samples probed with MTP18 antibody, and GAPDH antibody as an internal loading control (4 μg total protein loaded per lane). Images taken from the same blot, sequentially probed with antibodies. Full length blots available in Supplementary Fig. 2. (**c**) Western blot results graphed as densitometry values of MTP18 bands normalized to GAPDH, lines indicate paired control to crush samples. (**d**) Representative images of mTurquoise labeled mitochondria in P4 RGC neurites after AAV2 Anti-MTP18-mitochondrial(mito.)mTurquoise expression, co-labeled with MitoTracker-CMXRos. See Supplementary Fig. 3 for confirmation of protein knockdown/expression of MTP18 by viral vectors. (**e**) A representative maximum projection confocal microscopy image of a whole mounted retina with mitochondrial labeling after intravitreal injection of AAV2 Anti-MTP18-(mito.)mTurquoise virus. Mitochondrial labeling is visible within the nerve fiber layer, RGC layer, and segments of the inner plexiform layer. White arrows are pointing to fasciculated RGC axons running towards the optic nerve head. (**f**) Mean mitochondrial volume of mitochondria identified in fasciculated RGC axons in control and optic nerve crushed retinas, labeled by indicated knockdown or expression AAV2 virus. Data represented as a percent of the contralateral control retina, N ≥ 7 de-identified rats and images (significance determined by Student’s t-test within viral groups and one-way ANOVA with Tukey multiple comparisons between groups, *p ≤ 0.05). All error bars represent + SEM. (**g**) Representative 63x image of fasciculated RGC axons in an Anti-MTP18-(mito.)mTurquoise virus treated retina. Arrows highlight abnormal donut shaped mitochondria morphologies found in Anti-MTP18 treated axons.

Mitochondrial fission or suppressed fusion has been associated with early stages of neurodegeneration within the visual system^31^. Furthermore, given that KLF9 has been previously shown to increase in expression shortly after optic nerve injury^25^, we asked if MTP18’s expression plays a role in influencing mitochondrial dynamics after injury. To investigate this, we first confirmed that MTP18 is expressed at the protein level in adult (P40) RGCs by immunofluorescence, which showed elevated levels of MTP18 in the ganglion cell layer colocalizing with RBPMS, a selective marker of RGCs in the retina (Fig. 5a)^32^. We also detect the expression of MTP18 in adult retinas by western blot and quantify MTP18’s expression 24 and 48 hrs after crush, time points which reflect KLF9’s increased expression in RGCs^25^. At these time points we found that MTP18 expression increases after crush by approximately 20% at 24 hrs (detected in 2 out of 3 rats) and 50% at 48 hrs, relative to non-crushed contralateral controls and GAPDH (Fig. 5b,c), suggesting that MTP18 is involved in early responses to injury in RGCs. We also detected a more muted expression of MTP18 within the photoreceptor outer segment layer (Fig. 5a), which is known to be rich in mitochondria and could contribute to the overall MTP18 signal detected in blots, although the changes identified after optic nerve crush are likely from RGC responses, as this model is a selective and well established degeneration model for RGCs^33^.

We then asked whether MTP18 expression correlates with decreases in mitochondrial size and whether the modulation of MTP18 expression could alter mitochondrial size after optic nerve crush in RGCs. To measure mitochondrial size and express/knockdown MTP18 within the same injured RGCs *in vivo*, we applied AAV-2 vectors, as described above, to modulate MTP18 or luciferase (control) expression and simultaneously label mitochondria with mTurquoise, a blue shifted fluorescent protein. These vectors demonstrate an effective knockdown or expression of MTP18 at the RNA level (Fig. 4c) and at protein level (Supplemental Fig. 3), with anti-MTP18 virus reducing MTP18 protein levels by approximately 75% and MTP18 expression virus increasing levels by 250% as compared to anti-Luciferase virus-treated cells or untreated cells (Supplemental Fig. 3f). Here we used rat hippocampal neuron isolates, which also express MTP18 at baseline, and as demonstrated in our previous studies, provide increased cell and protein yields during western blotting or virus testing procedures while maintaining similar biochemical and viral responses as RGCs^11,25^. We also demonstrated that these viruses are capable of faithfully labeling mitochondria with limited loss in transduction efficiency, as demonstrated by mitotracker-mTurquoise colocalization in cultured RGCs (Fig. 5c,d) and uniform transduction in retinas after intravitreal injection (example image, Fig. 5e). In addition, these vectors are designed to express the mitochondrially targeted mTurquoise reporter with silencing RNAs or MTP18 at relatively comparable levels, with both expressed off of one polycistronic transcript or in the case of MTP18 via a PTA linker sequence^25,30^.

Applying these vectors in our optic nerve crush injury model, we find that control (luciferase knockdown) treated RGCs experience a significant decrease in mitochondrial size within retinal axons at 48 hrs post crush, as compared to contralateral sham surgery controls (Fig. 5f). We also find that MTP18 expression does not lead to a further reduction in mitochondrial size as compared to crush-control RGC axons. However, in MTP18 knockdown treated RGCs, mitochondrial size fails to decrease in the presence of crush injury, as compared to all other crush conditions. Thus, our data suggest that increased MTP18 expression is a component in reducing mitochondrial size concomitant with optic nerve crush, and that suppressing MTP18 expression is sufficient to overcome crush induced mitochondrial size decreases. Interestingly, in MTP18 knockdown treated RGCs we also see atypical mitochondrial morphologies in the shape of donuts within RGC axons after crush (Fig. 5g), which is suggestive of increased “end-to-end/side-to-end fusion”, increased mitochondrial volume, and is proposed to be a safeguard for increased volume tolerance and mitochondrial machinery^34–36^.

### MTP18 knockdown promotes increased neurite outgrowth

**Figure 6:**
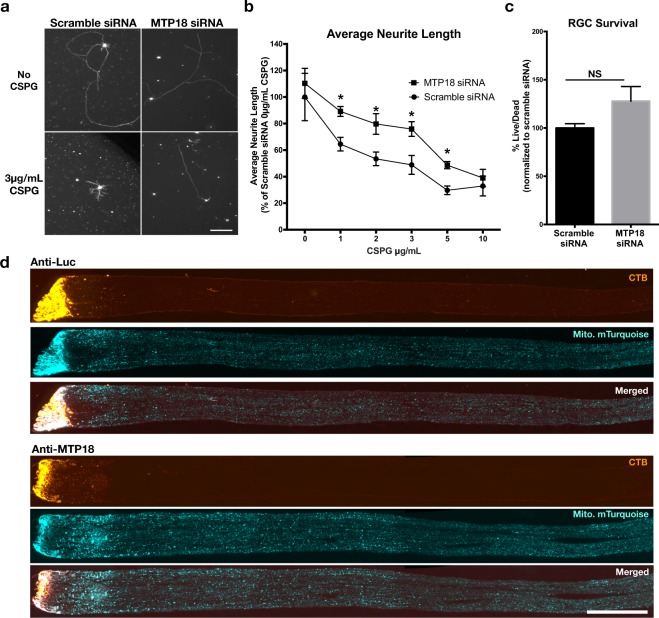
MTP18 knockdown promotes neurite outgrowth on inhibitory substrate but is not sufficient for promoting axon regeneration after optic nerve crush injury. (**a**) Representative images of P4 RGCs neurite growth after electroporation with a non-targeting scramble siRNA or MTP18 targeting siRNA and seeding on PDL + Laminin or PDL + Laminin + CSPGs (3 μg/ml) for 72 hrs (scale bar 100 μm). (**b**) Average neurite length of P4 RGCs electroporated with scramble siRNA or MTP18 siRNA and seeded onto different concentrations of CSPGs. All points are N = 4 repeat electroporation experiments per point, normalized to average neurite length of scramble siRNA seeded onto PDL + Laminin only treated wells (significance between scramble and MTP18 siRNA treatments at each CSPG concentration was determined by Student’s t-test, *p ≤ 0.05). (**c**) Scrambled and MTP18 siRNA-electroporated RGCs were plated at the same density as in neurite outgrowth experiments and were assayed for living and dead cells, using calcein AM (green labeled live cells) and sytox (orange labeled dead cells). Tiled images were captured per well and green and red cells were counted and graphed as percent ratio, live over dead cells, normalized to scrambled siRNA controls. (N = 4 repeat electroporation experiments, significance tested for by Student’s t-test, p ≥ 0.05.) See Supplementary Fig. 4a for representative calein Am and Sytox images. (**d**) Representative fluorescent images of P40 rats intravitreally injected with AAV2 Anti-MTP18 or Anti-luciferase virus prior to optic nerve crush. CTB Alexa-555 labeled axons after optic nerves crush show no significant regeneration. Mitochondrial (mito.) mTurquoise labeling from prior virus transduction and merged images with CTB, show degenerate labeling past the crush site and bright linear labeling of some preserved axons, just prior to the crush site (left side of images, scale bar 500 μm). See Supplementary Fig. 4b,c for RGC survival after optic nerve crush.

Since our previous data showed that increasing fusion or suppressing fission promotes RGC neurite outgrowth on CSPGs^10^, we hypothesized that knocking down MTP18, which the data suggests promotes more fusion, would also improve neurite extension on CSPGs. To test this hypothesis, we purified RGCs and electroporated them with an siRNA against MTP18 (mimicking the target sequence from our knockdown vector) or a control scramble-siRNA, followed by survival quantification and neurite tracing at 72 hrs of growth on CSPGs. This approach allowed us to assay the immediate effect of MTP18 knockdown on survival and neurite growth, unlike viral approaches which often require 3 or more days to express their payloads in RGCs^37^. Using this approach, we find that RGCs experience greater average neurite lengths with MTP18-siRNA treatment, as compared to scramble-siRNA treated RGCs on 3 and 5ug/mL of CSPGs (Fig. 6a,b), with no significant change in the survival between these conditions (Fig. 6c). Given these results and the concordant downregulation of MTP18 with KLF7 expression or KLF9 knockdown, previously shown effectors of axon regeneration^11,25^, we then asked whether MTP18 was acting downstream of KLFs’ to regulate axon regeneration *in vivo*. However, imaging of optic nerve axons after optic nerve crush revealed no significant regenerative capacity or survival of RGCs in MTP18 knockdown virus treated conditions, as evident by the degenerate mTurquoise signal from prior viral transduction in axons, the lack of CTB labeling past the crush site (Fig. 6d), and no significant improvement in RGC density in whole mount retinas (Supplemental Fig. 4).

## Discussion

In CNS neurons, higher levels of fission or fragmentation of mitochondria are often stereotyped as an early response after injury, preceding axon degeneration and cell death^38^. Here we add to these findings and demonstrate that MTP18, a previously unexplored fission regulator within the CNS, is a necessary and limiting regulator of mitochondrial fission during injury responses. Knockdown of MTP18 decreases mitochondrial fragmentation after optic nerve crush despite the injury being a pro-fragmentation cellular event. Although we show that MTP18 is rate limiting factor we cannot definitively claim whether MTP18 alone is sufficient to produce this pro-fission response, since overexpressing MTP18 under crush conditions doesn’t promote mitochondrial volume reduction beyond levels identified in control crush axons. It is also possible that MTP18 and/or coregulators of mitochondrial fission are performing at their maximum capacity after traumatic injury, making it difficult for us to identify any further pro-fission effects of MTP18 over other mitochondrial dynamics-modifying proteins. In addition, how MTP18 or mitochondrial fission/fusion balance contributes to axon growth is not well understood. MTP18 effects on axonal ATP or free calcium levels^39^, or signaling events normally scaffolded on mitochondria^40^ could be studied in related assays of mitochondrial size and axonal growth. Thus, further investigations will have to identify whether MTP18-regulated fission and mitochondrial ultrastructure is associated with such functional outcomes at mitochondria.

Regardless of MTP18’s hierarchical dominance over other pro-fission molecules, since inhibition of fission or increased fusion activity was previously shown to be favorable for axon growth^10^, we tested whether suppressing MTP18 expression could have a role in promoting axon growth in RGCs. Indeed, we find that acute knockdown of MTP18 increases neurite outgrowth on inhibitory CSPG substrates, which along with the data showing increased expression of MTP18 after optic nerve crush, is suggestive of an axon growth suppressive and/or axon degeneration roles for MTP18 in CNS trauma. However, *in vivo* studies failed to show any prominent axon regeneration or survival effects of MTP18 knockdown in RGCs experiencing optic nerve crush. MTP18 may need to potentiate other neuron-intrinsic axon growth regulators, or its expression may be more relevant to extrinsic glial-associated axonal repulsion cues, as suggested by MTP18’s mediation of axon growth inhibition by CSPGs *in vitro*. Combinatorial expression experiments could address whether MTP18 knockdown is necessary (but in our data not sufficient) to promote maximal regeneration. It will also be interesting to identify whether MTP18 contributes to pathological disease or whether there are any human diseases in which MTP18 mutations could be contributing factors, for example in photoreceptors or hippocampal degenerations, as suggested by our data showing MTP18 expression in these cells.

## Materials and Methods

### Animal use statement

Animal experiments were designed to conform with the ARVO statement for the Use of Animals in Ophthalmic and Vision Research and were reviewed and approved by the Stanford University Institutional Animal Care and Use Committee.

### Animal procedures

For optic nerve crush experiments P40 Sprague Dawley rats were anesthetized with xylazine (10 mg/kg, IP) and ketamine (80 mg/kg, IP). Optic nerves were then exposed from outer canthus behind the globe, and crushed for 5 seconds using #5 Dumont forceps (Fine Science Tools). The contralateral control eye received the same treatment but was spared from crush injury. In viral labeling experiments or for regeneration studies, rats received intravitreal injected of 4ul of virus approximately two weeks prior to crush injury at P28, to insure adequate viral expression. In regeneration studies, rats were allowed two additional weeks after crush injury for potential axon regeneration to occur. Then two days prior to sacrifice, rats received 4ul injections of cholera toxin subunit B-conjugated to AlexaFluor-555 (CTB-555 10 μg/μl; Thermofisher Scientific, C34776), to anterogradely label axons that have potentially regenerated past the crush site^11,25^. Rat were sacrificed by trans-cardiac perfusion with 4% PFA in PBS, and retinas and optic nerves were then post-fixed with 4% PFA for 1 hr at room temperature prior to immunostaining or sectioning. Rats experiencing any postoperative complications such as retinal ischemia or cataracts were removed from this study. All animals test conditions were masked from surgeons and tracked by numbered ear tags.

### Construct and RNAi design

Lenti virus expression components were cloned into pLenti-MP2 vectors (Addgene). All Lenti KLF plasmids contain the ORF of the human KLF sequence along with an c-terminal p2a linked mCherry tag. For the KLF7 vector VP16 was fused with the N-terminal of the KLF7 ORF to improve expression stability, as described elsewhere^12^. For AAV2 knockdown vectors, efficient knockdown was produced by cloning 4 concatenated shRNAs targeting luciferase, KLF9 (previously described^25^), or MTP18 (targeting sequences: 5′-TTAGGAACCAGGGAGCGGAAA-3′, 5′AGCTCAACCACACCACAGCTT-3′, 5′-TTGCCTTTGTCTATGGCATCG-3′, 5′-ATAGAGAGAGG CAGCACACAG-3′), into a SIBR cassette that drives the expression of these silencing RNAs and a downstream reporter via a single polycistronic transcript^30^. The AAV2 MTP18 expression vector contained the ORF for the RAT gene (NM_001006960), and KLF7 contained the ORF fused with the VP16 element, as in the Lenti virus iteration. In addition, AAV2 knockdown or expression plasmids contained a downstream or C-terminal P2A linked COX8a mitochondrial targeting sequence fused to the mTurquoise2 reporter^41^, to aid in fluorescent microscopy analysis of mitochondrial volume. All vector constructs were verified by sequencing prior to viral packaging. In neurite outgrowth studies, a validated siRNA against the rat MTP18 gene or a non-targeting scramble siRNA (Qiagen, SI01913191, SI03650318) was electroporated at 500 nM into 500k RGCs, prior to plating.

### Cell culture

RGCs were purified from male and female rat Sprague Dawley (Charles River Laboratories) pups at the indicated ages by immunopanning, and cultured on poly-D-lysine (10 μg/mL, Sigma Aldrich; P-6407) plus laminin coated (2 μg/mL, Sigma; L-6274) plates along with the addition of CSPGs for indicated neurite tracing experiments, in media as previously described^10,42^. For microarray or qPCR experiments, RGCs were plated at ~3–500 K in 6 well plates and treated with AAV2 or Lenti viruses for 5 days prior to RNA extraction and processing. For developmental time point microarrays RGCs were purified and directly processed for expression studies. In neurite out growth assays, RGCs were electroporated with siRNAs and cultured at low density ~5k cells per well in a 48 well plate for 72 hrs prior to antibody labeling and automated neurite tracing. For hippocampal neuron preps, Sprague-Dawley rat embryonic day 18 pups were sacrificed and CA1-CA3 regions were dissected in PBS with 10 mM D-glucose. Samples were digested with 0.05% trypsin-EDTA in PBS for 20 min at 37 °C, centrifuged at 200 g for 2 min, and then triturated with a fire-polished glass pipet in Hank’s balanced salt solution (HBSS) with calcium and magnesium. Dissociated neurons were plated on to poly-L-lysine coated plates in plating medium (10% v/v horse serum in DMEM). Four hours after plating, the medium was replaced with neurobasal medium supplemented with 2% B27, 0.5 mM L-glutamine and 1 mM pyruvate. Four days later, 4 μM arabinosyl cytosine was added to inhibit glial proliferation and cells were cultured for 2 weeks prior to viral labeling (labeling period is the same as with RGCs).

### Microscopy

For retinal imaging whole retinas were dissected, flat mounted onto a glass side (RGC layer facing up), placed within an adhesive spacer (Sigma, GBL654006), covered in prolong gold (Thermofisher Scientific, P36930), and coversliped. Images were then taken using a Zeiss LSM 880 confocal system with airyscan imaging mode, followed by airyscan processing using Zeiss Zen software. For mitochondrial volume rendering in optic nerve crush studies, images of RGC axons were acquired at 63x for each sample at a site approximately 100 um from the optic nerve head at axonal fasciculations within the nerve fiber layer (approximately a 10–15 um z-stack). All image analysis was performed using Volocity Imaging Software (Perkin Elmer), mean mitochondrial volume was rendered per image/retina (using the identify objects by intensity module) and consisted of >100 mitochondria. For viral transduction efficiency or RGC density assessment large area imaging studies of whole mounted retinas was conducted. 10x tiled image series were taken with ~60 um stacks and individual RGCs were identified and counted normalized per image area in Volocity or by manual counts in ImageJ, when cells were not easily detected by software. In *in vitro* hippocampal virus labeling or RGC survival studies, imaging was performed by wide field microscopy using a Zeiss Axio Observer. Cells were counted using ImageJ.

### Electron microscopy

To get slices through axonal sections, RGCs were purified as described and plated on aclar plastic inserts in a 48 well plate. Following seeding cells were treated with viruses to express and/or knockdown KLFs/MTP18 for 5 days. Then cells were fixed with one half Karnovsky’s fixative; 2.5% glutaraldehyde and 2% paraformaldehyde (PFA) in 0.2 M cacodylate buffer. Samples were then rinsed in 0.1 M phosphate buffer with osmium tetraoxide. Osmicated tissues were rinsed in 0.15 M phosphate buffer and dehydrated with graded concentrations of cold ethanol, ranging from 25 to 100%. Dehydrated samples were then rinsed with propylene oxide and embedded in Epon-Araldite with DMP-30 and sectioned onto grids for TEM imaging (All reagents were purchased from Electron Microscopy Sciences). Mitochondrial areas were calculated per mitochondria using ImageJ analysis software (National Institutes of Health). TEM images were acquired at 6000x magnification and axonal localized mitochondria were clearly distinguishable from vesicles, monofilaments and other internal cellular matter.

### Mitochondrial purification

Whole retinas were dissected from CO2 sacrificed rats, and homogenized using a dounce tissue grinder (Wheaton; 357538) with 30 strokes in mitochondrial isolation buffer (Miltenyi Biotec, 130-096-946) with protease inhibitors (Thermofisher Scientific, 78425). A small sample, ~100ul, was then pulled from the homogenate and was used for a total cell lysate fraction. Another fraction was pulled and spun at 20,000 g, to pellet organelle, and was used as the cytosolic fraction. The remaining sample was then centrifuged at 1000 × g and the supernatant was pulled for subsequent magnet based mitochondrial isolation according to Milteny Biotec’s Mitochondrial Isolation Kit. Isolated mitochondria were washed, pelleted, and resuspended in RIPA buffer. All other samples were also resuspended in RIPA buffer concentrate to make a final concentration of 1x RIPA. All procedures were performed on ice or at 4 °C, to preserve mitochondrial integrity.

### Neurite tracing

Cultured RGCs, described above, were fixed and permeabilized with 4% PFA and 0.2% Triton X in PBS, respectively. Tubulin was then labeled in RGCs using an anti-β tubulin antibody produced by E7 hybridoma (Developmental Studies Hybridoma Bank, Iowa City) at 1:1000 overnight at 4 °C, along with 2ndary antibody labeling at 1:500 for 4 hours at room temperature (anti-mouse conjugated Alexa488; Thermofisher Scientific, A11029) and DAPI nuclei staining at 1:5000. RGCs average neurite outgrowth was then imaged and quantified using automated microscopy via an ArrayScan VTI system and Cellomics Neuronal Profiling BioApplication image analysis software (Thermofisher Scientific), as previously described^11,12,25,26^.

### *In vitro* survival

RGC survival was measured in electroporation experiments, by labeling cells with Hoechst 3334 (4 μM; Thermofisher Scientific, H3570), calcein AM (2 μM; Thermofisher Scientific, C1430) and SYTOX orange (1 μM; Thermofisher Scientific, S11368) for 10 min in full media, washed with full media, and imaged in full media. Tiled 10x images were acquired on a Zeiss wide field microscope (Zeiss Axio Observer) and scored in ImageJ. All calcein AM positive-SYTOX negative neurite extending cells were scored as live and all calcein AM negative–SYTOX positive cells were scored as dead RGCs^43^.

### Western blots

To evaluate the isolated mitochondria and other homogenate fractions, samples were analyzed by SDS page using tris-glycine gels and transferred onto PVDF for subsequent blotting procedures (Biorad, 4561094 and 1704156). Antibodies used for blotting procedures included an antibody against MTP18 (Abcam, ab198217), Complex IV subunit I (MTCO1; Abcam, ab14705), and GAPDH (Cell Signaling Technology, 2118 S). An MTP18 positive control protein lysate was used in blots to confirm the detection of MTP18 (Santa Cruz, sc-125655). Prestained protein ladders were loaded in all gels and were used to identify the molecular weight of proteins (Biorad, 1610375, and Thermofisher Scientific, 26617). All blotting procedures with primary antibodies were carried out overnight, at 4 °C in 5% milk in a T(0.1%)-TBS solution. All secondary antibodies HRP conjugated were incubated for 4 hr (1:5000) at room temp, prior to ECL activation and imaging procedures (General Electric, NA931, NA9340, Amersham Imager 680). Loading normalization and band quantification was conducted using the ImageJ gel analyzer tool.

### Immunostaining

Retinal flat mounts, after fixation and dissection, were rinsed in PBS and blocked-permeabilized for 1 hr with 20% goat serum and 0.4% Triton X-100, in antibody buffer containing 150 mm NaCl, 50 mm Tris base, 1% BSA, 100 mm l-lysine, and 0.04% Na azide, pH 7.4. Retinas were then incubated overnight at 4 °C in antibody buffer containing primary antibodies, washed with PBS 5 times, incubated with secondary antibodies for 1 hr, and with DAPI for 10 min in antibody buffer at room temperature. Samples were then washed again 5 times with PBS before mounting in Prolong Gold (Thermofisher Scientific, P36930). The same procedure was carried out for sectioned retinas and optic nerves, except that prior to permeabilization and staining, samples were embedded in OCT and 15 μm frozen sections were placed on glass slides. Primary antibodies used for immunostaining included anti-MTP18 (1:100; Abcam, ab198217), anti-Brn3a (1:100; Millipore, MAB1585) and anti-RBPMS (1:1000: PhosphoSolutions, 1832-RBPMS). Secondary antibodies were Alexa Fluor 488-, 555-, or 647-conjugated, highly cross-adsorbed antibodies (1:500; Thermofisher Scientific).

### RNA expression

To detect gene expression changes, RNA was extracted from isolated and cultured RGCs using the RNeasy Plus Micro Kit (Qiagen, 74034). RNA isolates were then processed shipped to the Neuroscience Microarray Consortium (UCLA), where they were processed for rat genome arrays (Affymetrix, GeneChip Rat Genome 230 2.0 Array)^26^. Raw data files were normalized using the quantile method with GeneSpring GX 11 software (Agilent Technologies). Normalized data were filtered as described above (raw data files available at the NIH GEO Database - Series ID # GSE92507, analyzed data in Supplemental Dataset 1). For RT-qPCR experiments, RNA extracts were reverse transcribed (Biorad, 1708891) and quantified for KLF, MTP18, and GAPDH expression using Taqman probes (Thermofisher Scientific. 4369542, Rn00589498_m1, Rn01461756_g14453320, Rn99999916_s1), and data was acquired on QuantStudio7 Flex Real-Time PCR System (Thermofisher Scientific).

### Statistics

Statistical analysis was done in Prism (Graphpad). To compare quantitative variables, between two samples two-tailed Students t-test was conducted, for more than two sample comparisons ANOVA and post-hoc t-tests were done with a *p*-value ≤ 0.05 indicating statistical significance.

## Supplementary information

Supplementary Figures 1-4

Dataset 1
