# Supplementary material for: MTP18 is a Novel Regulator of Mitochondrial Fission in CNS Neuron Development, Axonal Growth, and Injury Responses
Source: Sci Rep. 2019 Jul 23;9:10669. doi: 10.1038/s41598-019-46956-5 (PMC6650498; doi:10.1038/s41598-019-46956-5)
Supplement: Supplementary file 1 — Supplementary Figures 1-4 [file 41598_2019_46956_MOESM1_ESM.pdf]

MTP18 is a Novel Regulator of Mitochondrial Fission in CNS Neuron Development, Axonal Growth, and Injury Responses

Alex Kreymerman[1,2], David N. Buickians[1], Michael M. Nahmou[1], Tammy Tran[3], Joana Galvao[1], Yan Wang[3], Nicholas Sun[3], Leah. Bazik[3], Star K. Huynh[1], In-Jae Cho[1], Tomasz Boczek[1], Kun-Che Chang[1], Noelia J. Kunzevitzky[1], Jeffrey L. Goldberg[1*]

1. Byers Eye Institute and Spencer Center for Vision Research, Stanford University, Palo Alto, CA 94303
2. University of Miami Miller School of Medicine, Miami, FL 33136
3. University of California, San Diego, CA 92093

Address correspondence to:
Alexander Kreymerman (akreymer@stanford.edu)
Jeffrey L. Goldberg (jlgoldbe@stanford.edu)
Byers Eye Institute
Stanford University
2452 Watson Ct
Palo Alto, CA 94303

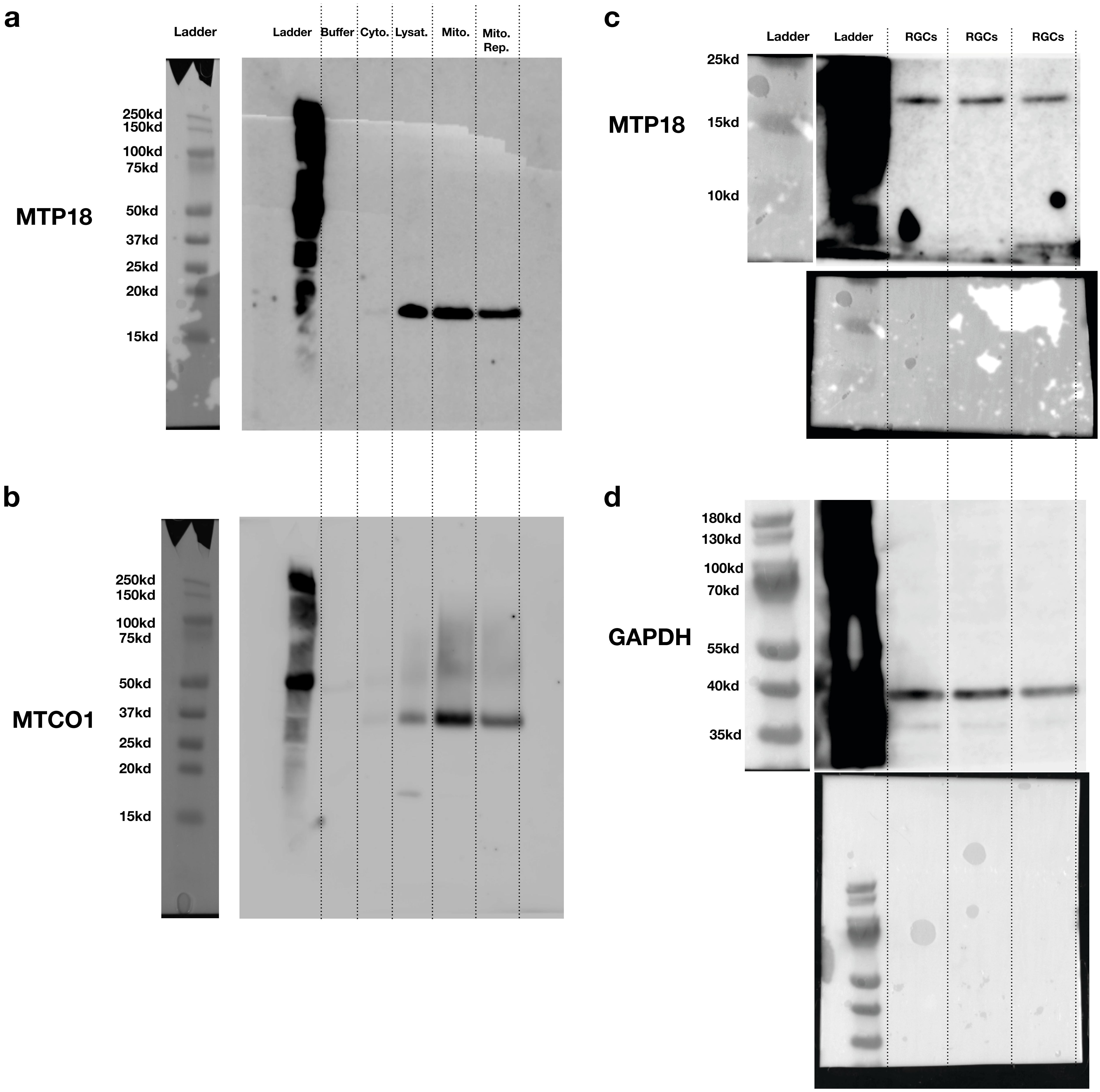

Supplementary Figure 1

Images of the entire membrane from western blots of RGC lysates and mitochondrial enrichment (cropped images shown in Fig. 4a and b). Samples loaded as presented in the images. (**a**) Loading order; ladder, buffer blank, cytosolic fraction (cytosolic), crude total cell lysate fraction (lysate), and purified mitochondria fraction (mito.) along with a second repeat mito. fraction (mito. rep.). Both images (**a**) and (**b**) are from the same membrane blotted sequentially first for MTP18 and then for MTCO1. White light ladder images (Precision Plus Protein Kaleidoscope Prestained Protein Standards, on the left). (**c)** Loading order; ladder and 3 replicate P4 RGC purification cell lysates. Images (**c**) and (**d**) are from the same membrane cut at 25kd and blotted in parallel with MTP18 and GAPDH. White light ladder images (PageRuler Prestained Protein Ladder, on the left) are provided from a cropped image of the whole membrane shown below each blot in c and d.

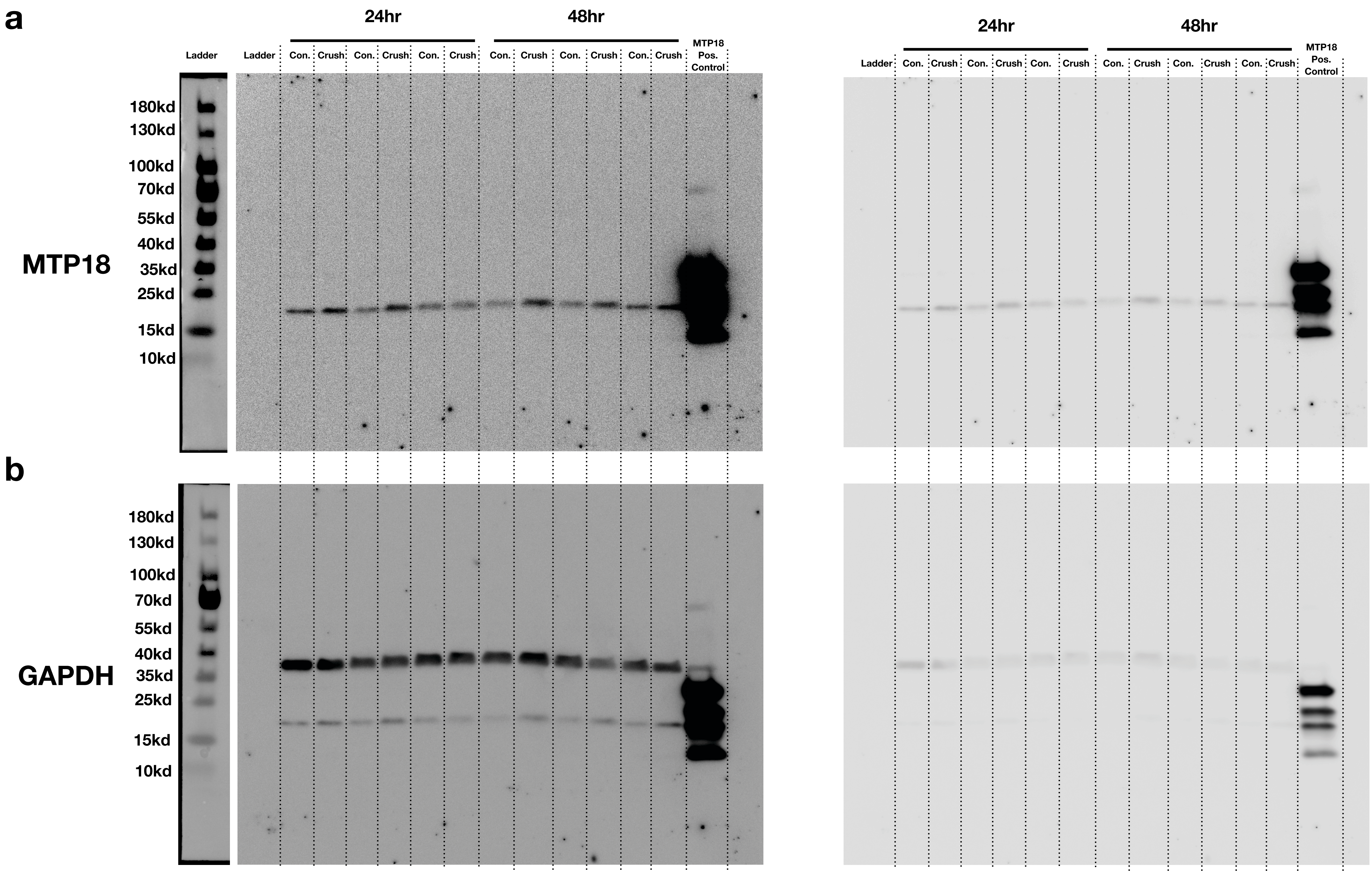

Supplementary Figure 2
Images of the entire membrane from western blots showing MTP18 expression in retinas after optic nerve crush. Sample loading patterns are presented in the images as in figure 5 a, ladder and sequential control vs crush comparisons at 24 and 48hr times points. A positive control was added for MTP18 detection (MTP18 pos. control), consisting of lysate from 293T cells over-expressing MTP18. The same membrane was initially probed with MTP18 antibody (**a**), and then GAPDH antibody (**b**) to serve as an internal loading control. Remnant banding patterns from MTP18 antibody staining are visible in (**b**), as both MTP18 and GAPDH antibodies were probed with rabbit secondaries. Thresholded images on the left are non-cropped versions of the image presented in figure 5a, the images on the right are of the same membranes but without image enhancement/thresholding. These images were used to quantify MTP18 and GAPDH banding densitometry. White light ladder images (PageRuler Prestained Protein Ladder, on the left) are provided from a cropped image of the whole membrane.

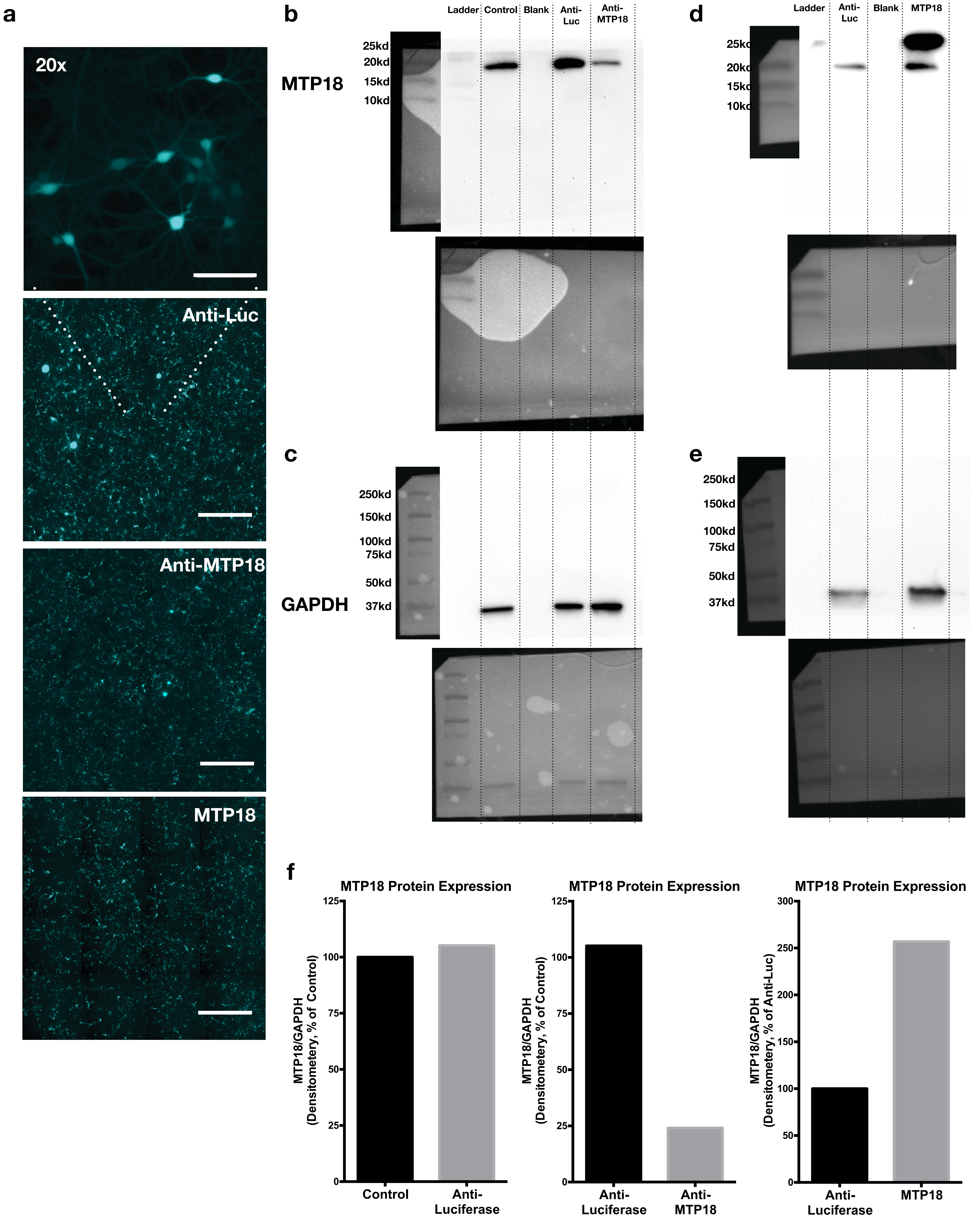
a
20x
Anti-Luc
Anti-MTP18
MTP18
b
Ladder
Control
Blank
Anti-Luc
Anti-MTP18
MTP18
25kd
20kd
15kd
10kd
d
Ladder
Anti-Luc
Blank
MTP18
c
250kd
150kd
100kd
75kd
50kd
37kd
GAPDH
e
250kd
150kd
100kd
75kd
50kd
37kd
f
MTP18 Protein Expression
MTP18/GAPDH (Densitometery, % of Control)
Control
Anti-Luciferase
MTP18 Protein Expression
MTP18/GAPDH (Densitometery, % of Control)
Anti-Luciferase
Anti-MTP18
MTP18 Protein Expression
MTP18/GAPDH (Densitometery, % of Anti-Luc)
Anti-Luciferase
MTP18

Supplementary Figure 3

AAV2 virus efficiency testing for MT18 knockdown and expression in CNS neurons. (**a)** 20x zoomed image of virally labeled hippocampal neurons (scale bar 50 µm) and corresponding 5x tiled regions (scale bar 2000 µm) of hippocampal cells transduced with the indicated AAV2 virus, showing efficient expression and homogenous transduction by viruses. (**b-e)** Images of the entire membrane from western blots of hippocampal lysates. Samples loaded as presented in the images. (**b**) Shows that control untreated hippocampal cells express MTP18, and that anti-MTP18 virus treated cells can reduce MTP18 expression relative to anti-Luciferase treated cells and (**c**) GAPDH loading controls. (**d**) Increased MTP18 expression in MTP18 expression virus-treated neurons relative to anti-Luciferase-treated cells. Here two bands are visible in the MTP18 expression vector-treated neurons. The slightly elevated band (shifted by approximately 5kd) is the virally expressed MTP18, and is the result of additional amino acids in the n-terminus and c-terminus during cloning of a P2A linkage sequence and Flag tag, cloning sites previously shown not to compromise MTP18 activity[23,24]. (**e**) Corresponding GAPDH loading controls are shown. All images from the same membrane cut and blotted in parallel with MTP18 and GAPDH. White light ladder images are provided from a cropped image of the whole membrane shown below each blot. (**f**) Blots were quantified by ImageJ and MTP18 expression was graphed relative to GAPDH banding densitometry for the labeled samples.

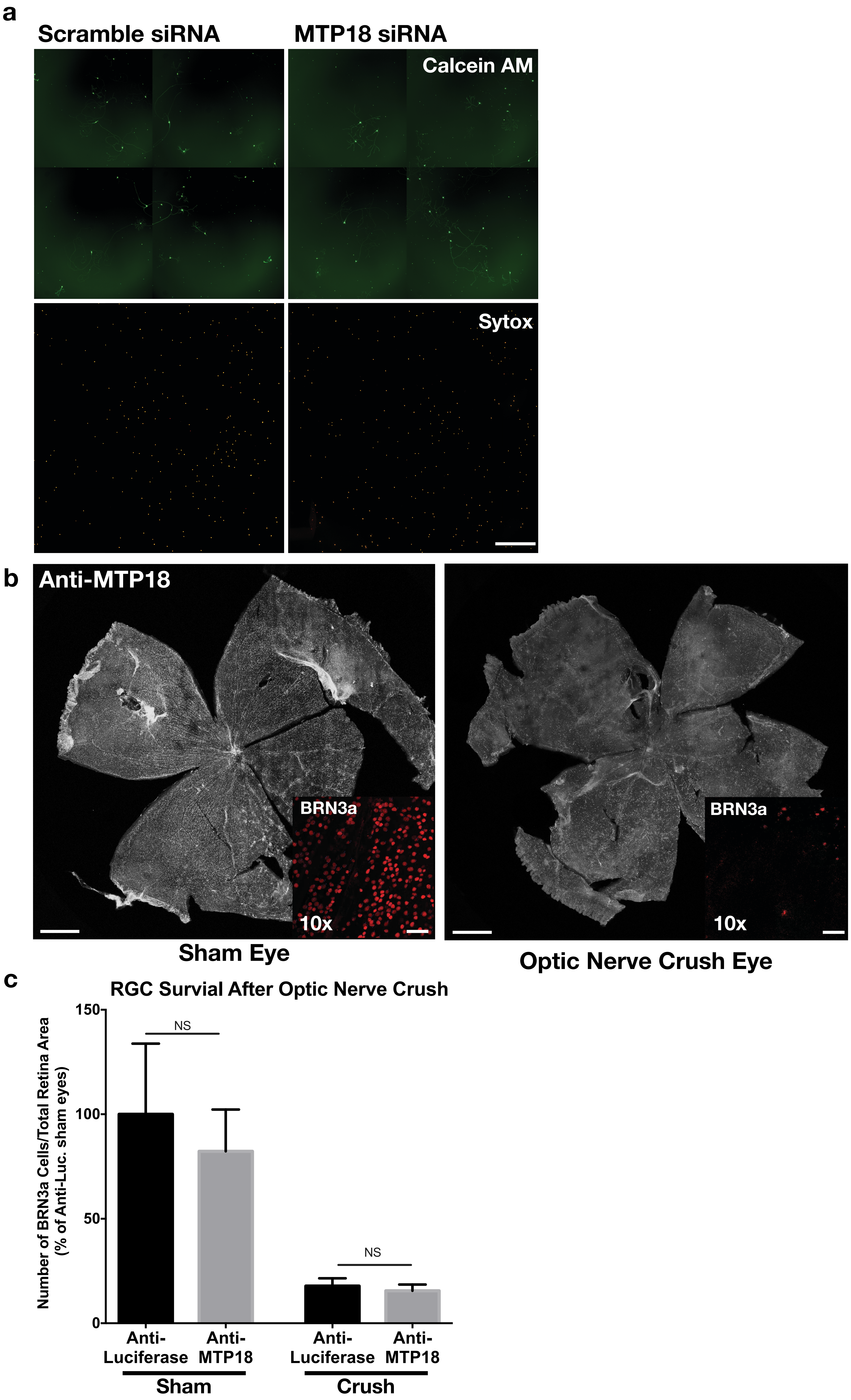

a
Scramble siRNA
MTP18 siRNA
Calcein AM
Sytox
b
Anti-MTP18
BRN3a
10x
BRN3a
10x
Sham Eye
Optic Nerve Crush Eye
c
RGC Survial After Optic Nerve Crush
Number of BRN3a Cells/Total Retina Area (% of Anti-Luc. sham eyes)
150
100
50
0
NS
NS
Anti-Luciferase
Anti-MTP18
Anti-Luciferase
Anti-MTP18
Sham
Crush

Supplementary Figure 4

Representative images of in vitro and in vivo RGC survival after MTP18 knockdown and/or optic nerve crush. (**a**) Representative tiled image fields used for graphed values in Fig. 6c. RGCs labeled with calcein AM (labeling viable cells green) and sytox orange (labeling dead or dying cells orange), 72hrs after electroporation of the indicated siRNAs (scale bar 500 μm). (**b**) Representative tiled images of whole mounted rat retinas from an Anti-MTP18 treated animal that received optic nerve crush (scale bar 1000 μm), as in Fig. 6d. Sham procedure control eyes and contralateral optic nerve crush eye. BRN3a staining, a marker of ganglion cells, reveals typical ganglion cell distribution and density within uncrushed eyes and a significant decrease in RGC density after optic nerve crush, zoomed in sub-panels provided for better visualization (scale bar 50 μm). (**c**) Counting the number of Brn3a positive ganglion cells, normalized to total retinal area, and graphed as a percent relative to Anti-Luciferase treated sham retinas, reveals that MTP18 knockdown doesn't significantly change the proportion of surviving ganglion cells when compared to Anti-Luciferase treatments within sham or crushed eye groups. Although, as expected, significance was detected between uncrushed and crushed retinas, indicating a successful crush procedure. N of 4 rats per sham vs crush group, 8 retinas total (significance determined by a one-way ANOVA with Tukey multiple comparisons within and between groups).
